# Dual-layer detector spectral CT-based machine learning models in the differential diagnosis of solitary pulmonary nodules

**DOI:** 10.1038/s41598-024-55280-6

**Published:** 2024-02-25

**Authors:** Hui Lu, Kaifang Liu, Huan Zhao, Yongqiang Wang, Bo Shi

**Affiliations:** 1 School of Medical Imaging, Bengbu Medical University, Bengbu, 233030 China; 2 Department of Radiology, Nanjing Medical University Affiliated Cancer Hospital, Jiangsu Cancer Hospital, Jiangsu Institute of Cancer Research, Nanjing, 210000 China

**Keywords:** Machine learning, Dual-layer detector spectral CT, Solitary pulmonary nodules, Logistic regression, Cancer, Diseases, Oncology

## Abstract

The benign and malignant status of solitary pulmonary nodules (SPNs) is a key determinant of treatment decisions. The main objective of this study was to validate the efficacy of machine learning (ML) models featured with dual-layer detector spectral computed tomography (DLCT) parameters in identifying the benign and malignant status of SPNs. 250 patients with pathologically confirmed SPN were included in this study. 8 quantitative and 16 derived parameters were obtained based on the regions of interest of the lesions on the patients’ DLCT chest enhancement images. 6 ML models were constructed from 10 parameters selected after combining the patients' clinical parameters, including gender, age, and smoking history. The logistic regression model showed the best diagnostic performance with an area under the receiver operating characteristic curve (AUC) of 0.812, accuracy of 0.813, sensitivity of 0.750 and specificity of 0.791 on the test set. The results suggest that the ML models based on DLCT parameters are superior to the traditional CT parameter models in identifying the benign and malignant nature of SPNs, and have greater potential for application.

## Introduction

Lung cancer, as a common cancer, has the highest mortality rate in the world^1^. Lung cancer usually manifests as solitary pulmonary nodules (SPNs) in the early stage, that is, round or quasiround lung parenchyma lesions with a diameter of no more than 3 cm and without other abnormalities^2–4^. Clinically, doctors usually adopt different treatment methods according to the benign and malignant characteristics of patients with SPNs. For benign SPNs, clinical observation or surgical resection of part of the tissue can be performed^5,6^, while for malignant SPNs diagnosed as high-risk, lobectomy or systemic lymph node dissection is necessary due to the possibility of postoperative recurrence and metastasis^7^. Therefore, the accurate preoperative identification of the benign and malignant status of SPNs is clinically important in guiding the treatment of patients.

The complexity of human physiology and pathology has been shown to be predicted using artificial intelligence algorithms. Several studies have demonstrated the efficacy of machine learning (ML) models based on computed tomography (CT), magnetic resonance imaging (MRI) and positron emission tomography/CT (PET/CT) imaging features in assessing the benignity and malignancy of SPNs^8–10^. However, the cost of PET/CT examination is high, which will bring economic burden to patients^11^; MRI examination takes a long time, and the image artifacts appear due to the cardiopulmonary movement of the human body, which affects the accuracy of the results^12^; CT is widely used in pulmonary imaging, but the quantitative diagnostic information provided by traditional CT is limited, which may ignore some smaller lesions^13^.

In recent years, with the development of image inspection equipment, dual-layer detector spectral CT (DLCT) can not only provide traditional CT images in a single scan, but also obtain virtual noncontrast (VNC) maps, virtual monoenergetic images (VMI), effective atomic number (Zeff) maps and other multi-parameter imaging, which has been widely used in the quantitative analysis of multiple clinical organ systems^13,14^. In addition, it has been reported that DLCT analysis combined with ML can achieve higher accuracy in lesion classification than traditional CT^15^. Wen et al. and Zhang et al.’s study also showed that DLCT parameters such as spectral curve slope (λ_HU_) and monoenergetic CT values of 40 keV (CT_40 keV_) can effectively distinguish benign and malignant SPNs^16,17^. However, their study had a small sample size and did not build ML models for further study. Therefore, in this study, we construct ML models characterized by DLCT quantitative and derived parameters to verify whether it can further improve the identification of benign and malignant SPNs.

## Results

### Participant characteristics

The clinical data and preoperative DLCT parameters of 250 patients with SPN were collected in this retrospective study, of which 33 cases were pathologically diagnosed as benign and 217 cases were malignant. The results of the univariate analysis of the clinical characteristics of patients (including sex, age and smoking history) are shown in Table 1. Age was significantly different between patients with benign and malignant SPNs (*P* = 0.023), whereas sex and smoking did not show a significant difference between benign and malignant SPNs (*P* = 0.630, *P* = 0.738).

**Table 1:** Comparison of clinical data of all patients.

Characteristics	Benign SPNs (N = 33)	Malignant SPNs (N = 217)	*P* value
Age (years)	62.0 [56.0, 66.0]	66.0 [57.0, 71.0]	0.023*
Sex			0.630
Male	18 (54.5%)	128 (59.0%)	
Female	15 (45.5%)	89 (41.0%)	
Smoking			0.738
No	17 (51.5%)	105 (48.4%)	
Yes	16 (48.5%)	112 (51.6%)	

Values are expressed as median [interquartile ranges] or the number of patients (percentages). *P* value less than 0.05 (marking with *) indicates the data are statistically significant.

Table 2 shows the DLCT parameters of the patients. The univariate analysis results showed that the DLCT parameters with significant differences between benign and malignant SPNs included diameter, CT values of lesions on VNC images (CT_SPN_VNC_), the Zeff, iodine concentration and electron density of lesions (Zeff_SPN_, IC_SPN_ and ED_SPN_), CT values of lesions on monoenergetic images (CT_SPN_40 keV_, CT_SPN_70 keV_), the ratio of the lesion to the aorta on VNC images (SAR_VNC_), the ratio of the lesion to the aorta on monoenergetic images (SAR_40 keV_ and SAR_70 keV_), the difference between the enhanced lesion and the VNC lesion (Δ_40 keV_, Δ_70 keV_), the difference between the enhanced lesion and enhanced aorta on 40 keV monoenergetic image (Δ_SA_40 keV_), the ratio of Δ_40 keV_ or Δ_70 keV_ to the difference between the enhanced aorta and VNC aorta (NEF_40 keV_, NEF_70 keV_), λ_HU_, the normalized IC (NIC), the normalized ED (NED) and the normalized Zeff (NZeff). The DLCT parameters that did not differ significantly between the two groups of SPN patients were the calcium suppression of the lesion (CaS_SPN_), the normalized CaS (NCaS), the difference between the enhanced lesion and enhanced aorta on 70 keV monoenergetic image (Δ_SA_70 keV_), the ratio of Δ_40 keV_ or Δ_70 keV_ to the CT values of VNC lesions (CER_40 keV_, CER_70 keV_).

**Table 2:** Comparison of DLCT parameters in SPN patients.

Characteristics	Benign SPNs (N = 33)	Malignant SPNs (N = 217)	*P* value
Diameter (mm)	14.90 [11.45, 18.15]	20.00 [16.25, 24.50]	< 0.001*
CT_SPN_VNC_ (HU)	28.0 [21.0, 41.0]	36.6 [31.0, 40.9]	0.013*
Zeff_SPN_	8.0 [7.7, 8.2]	8.2 [8.1, 8.4]	< 0.001*
IC_SPN_ (mg/ml)	1.2 [0.7, 1.5]	1.6 [1.3, 1.9]	< 0.001*
ED_SPN_ (%EDW)	103.0 [102.1, 104.2]	103.8 [103.3, 104.3]	0.003*
CaS_SPN_	1.500 [− 5.7, 14.1]	− 0.2 [− 12.7, 7.1]	0.086
CT_SPN_40 keV_ (HU)	133.1 [81.3, 166.7]	170.3 [152.3, 205.6]	< 0.001*
CT_SPN_70 keV_ (HU)	58.7 [40.6, 71.7]	76.5 [68.9, 86.9]	< 0.001*
SAR_VNC_	0.6 [0.5, 0.9]	0.8 [0.7, 0.9]	0.004*
SAR_40 keV_	0.6 [0.4, 0.7]	0.8 [0.6, 0.9]	< 0.001*
SAR_70 keV_	0.3 [0.2, 0.3]	0.4 [0.3, 0.4]	< 0.001*
Δ_40 keV_ (HU)	103.2 [60.2, 126.9]	133.9 [114.4, 170.2]	< 0.001*
Δ_70 keV_ (HU)	30.7 [17.9, 37.6]	39.9 [34.1, 50.6]	< 0.001*
Δ_SA_40 keV_ (HU)	− 95.2 [− 127.6, − 58.6]	− 51.7 [− 95.3, − 8.6]	< 0.001*
Δ_SA_70 keV_ (HU)	− 166.1 [− 183.3, − 132.1]	− 149.9 [− 185.7, − 108.8]	0.093
CER_40 keV_	3.6 [2.5, 4.8]	3.8 [3.1, 5.4]	0.125
CER_70 keV_	1.1 [0.7, 1.4]	1.1 [0.9, 1.6]	0.084
NEF_40 kev_	0.5 [0.3, 0.7]	0.7 [0.6, 1.0]	< 0.001*
NEF_70 kev_	0.12 [0.1, 0.2]	0.22 [0.18, 0.30]	< 0.001*
λ_HU_	2.4 [1.4, 2.9]	3.1 [2.7, 3.9]	< 0.001*
NIC	0.16 [0.11, 0.20]	0.22 [0.17, 0.30]	< 0.001*
NED	0.97 [0.96, 0.98]	0.98 [0.97, 0.99]	0.013*
NZeff	0.78 [0.75, 0.80]	0.79 [0.76, 0.84]	0.008*
NCaS	− 0.02 [− 0.11, 0.04]	0.0 [− 0.07, 0.09]	0.137

Values are expressed as median [interquartile ranges]. *P* value less than 0.05 (marking with *) indicates the data are statistically significant.

### Diagnostic performance of the six ML models

**Figure 1:**
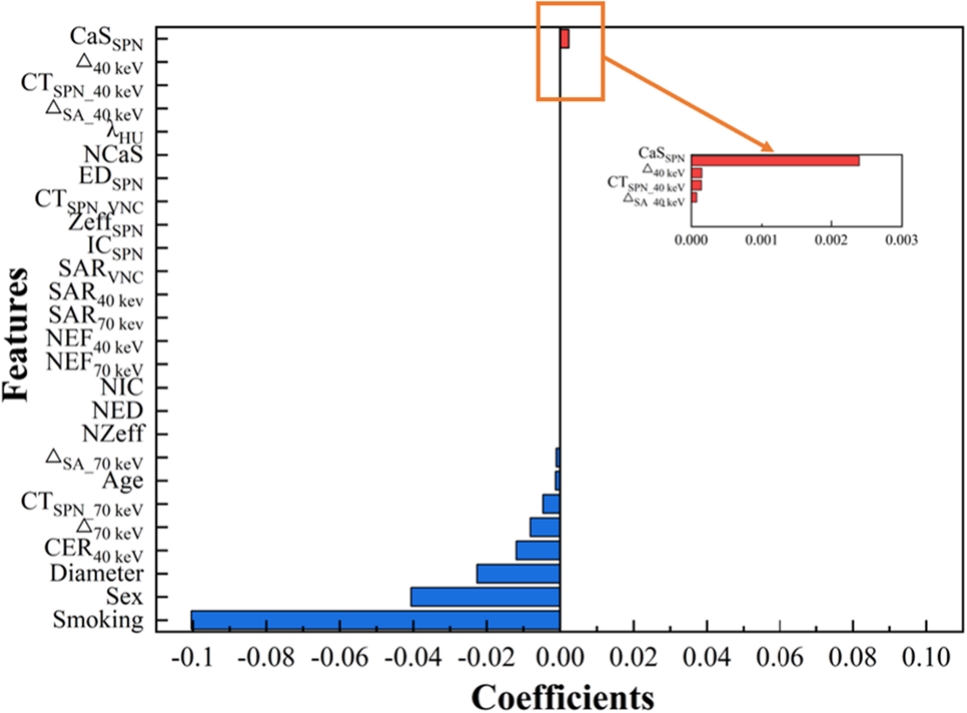
Features selected by LASSO with their estimated coefficients.

From 3 clinical parameters and 24 DLCT parameters, 10 parameters were selected as the input features of the ML models by the least absolute shrinkage and selection operator (LASSO) algorithm (Fig. 1). These features were CER_40 keV_, Δ_SA_70 keV_, Δ_70 keV_, CT_SPN_70 keV_, CT_SPN_40 keV_, CaS_SPN_, diameter, smoking, sex and age, respectively.

**Figure 2:**
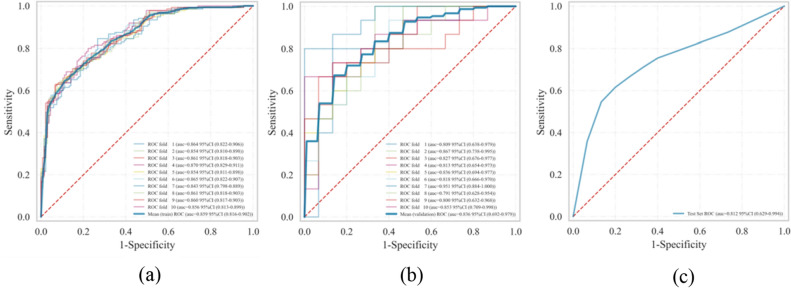
The ROC of the LR model. (**a**–**c**) represent the training set, validation set and test set respectively.

Table 3 shows the specific performance indicators of the 6 ML models in the training set and validation set after tenfold cross-validation, and Table 4 shows the evaluation indicators of the 6 ML models in the test set. It can be seen from Tables 3 and 4 that although the extreme gradient boosting (XGBoost), adaptive boosting (AdaBoost) and random forest (RF) models have higher areas under the receiver operating characteristic curve (AUC) in both the training and validation sets, their performance in the test set data was not ideal, and there may be overfitting phenomenon. In addition, the logistic regression (LR) model had the highest AUC (AUC = 0.812) in the test set, with accuracy, sensitivity and specificity of 0.813, 0.750 and 0.791, respectively, and therefore it can be assumed that the predictive performance of the LR model was better than the other 5 models (including Gaussian naive Bayes (GNB), support vector machine (SVM), XGBoost, RF, AdaBoost). Figure 2a,b shows the receiver operating characteristic curves (ROC) of the LR model for tenfold cross-validation in the training and validation sets. Where the blue solid line represents the average ROC curve, the red diagonal line indicates an AUC of 0.5, and the grey area represents the 95% confidence interval of the average ROC curve. Figure 2c shows the ROC curve of the LR model in the test set.

**Table 3:** Performance of 6 models after tenfold cross-validation.

Models	AUC	Accuracy	Sensitivity	Specificity
**LR**				
Training	0.859 (0.816–0.902)	0.774 (0.769–0.779)	0.693 (0.643–0.744)	0.862 (0.817–0.908)
Validation	0.836 (0.692–0.979)	0.743 (0.703–0.784)	0.820 (0.744–0.896)	0.787 (0.682–0.891)
**GNB**				
Training	0.859 (0.817–0.902)	0.779 (0.776–0.781)	0.699 (0.684–0.713)	0.866 (0.854–0.878)
Validation	0.848 (0.709–0.984)	0.773 (0.750–0.797)	0.773 (0.697–0.849)	0.860 (0.803–0.917)
**SVM**				
Training	0.819 (0.770–0.868)	0.742 (0.739–0.746)	0.730 (0.691–0.770)	0.761 (0.722–0.801)
Validation	0.813 (0.656–0.970)	0.713 (0.672–0.755)	0.707 (0.624–0.790)	0.873 (0.802–0.945)
**XGBoost**				
Training	1.000 (NaN–NaN)	0.996 (0.996–0.996)	1.000 (1.000–1.000)	1.000 (1.000–1.000)
Validation	0.974 (NaN–NaN)	0.897 (0.876–0.917)	0.947 (0.900–0.994)	0.967 (0.945–0.988)
**RF**				
Training	1.000 (NaN–NaN)	0.996 (0.994–0.997)	1.000 (1.000–1.000)	1.000 (1.000–1.000)
Validation	0.975 (NaN–NaN)	0.917 (0.899–0.934)	0.927 (0.877–0.976)	0.973 (0.952–0.995)
**AdaBoost**				
Training	0.999 (NaN–NaN)	0.986 (0.981–0.990)	0.989 (0.981–0.997)	0.990 (0.983–0.996)
Validation	0.909 (0.801–0.996)	0.847 (0.806–0.887)	0.880 (0.833–0.927)	0.907 (0.867–0.947)

NaN represents a null value. Values are expressed as mean (95% confidence interval).

**Table 4:** Performance indicators of 6 models in the test set.

Models	AUC (95% CI)	Accuracy	Sensitivity	Specificity
LR	0.812 (0.629–0.994)	0.813	0.750	0.791
GNB	0.705 (0.418–0.993)	0.840	0.750	0.851
SVM	0.784 (0.593–0.974)	0.787	0.750	0.821
XGBoost	0.789 (0.664–0.914)	0.827	1.000	0.537
RF	0.743 (0.511–0.976)	0.853	0.750	0.896
AdaBoost	0.761 (0.520–1.000)	0.853	0.625	0.925

CI stands for the confidence interval.

## Discussion

In this study, we evaluated the correlation between benign and malignant nodules and DLCT quantitative parameters and derived parameters in patients with SPN, and established 6 ML models based on DLCT parameters and patient clinical parameters, among which the LR model showed the best predictive performance (AUC = 0.812), suggesting that the application of ML models are valuable in distinguishing benign and malignant SPNs.

So far, most of the classification studies using ML algorithms have relatively high accuracy, which has also led to an increasing number of studies on ML models in identifying the benign and malignant pulmonary nodules. Table 5 listed some of the studies applying ML models to identify the benign and malignant pulmonary nodules, including different modelling methods such as conventional CT imaging features, CT radiomic features and PET/CT texture features. The studies by Ma et al.^18^ and Uthoff et al.^19^ built ML models based on conventional CT imaging features, and the study by Beig et al.^20^ built ML models based on CT radiomic features. From the perspective of model performance, the ML model based on CT radiomic features is slightly better than the models based on conventional CT features. The studies by Zhang et al.^21^ and Chen et al.^22^ are both ML models constructed based on PET/CT texture features. The ability of PET/CT to obtain both anatomical structure and functional information makes the model performance better than that of conventional CT. However, the high cost of PET/CT cannot make it widely applicable in the clinic. The ML models developed in this study are mainly based on extracted DLCT features. As a new type of energy spectrum CT, a variety of functional analysis tools enable DLCT to provide a variety of quantitative and derivative parameters under the premise that patients do not need to bear additional radiation doses, so as to quantify the essential characteristics of lesions and provide additional value for the classification and differentiation of tumor lesions. In this study, we constructed 6 different ML models based on the same features. Among them, XGBoost, RF and AdaBoost are integrated models composed of several weak classifiers, but overfitting occurs in the modeling process. Although these three models performed well in model training, they did not show good predictive performance when validated with test set data. This shows that integrated models do not always perform best in different classification studies. Among the other three ML models (LR, GNB and SVM), the LR model had the highest AUC, sensitivity and second-highest accuracy, making it the ML model with the best prediction performance. In terms of model performance, the LR model in this study is better than the models constructed with conventional CT features, but not as good as the ML models based on PET/CT features. On the one hand, this may be due to the fact that the features extracted are different because of the different imaging instruments used; on the other hand, it may be that the number of benign SPN patients and malignant SPN patients in this study was obviously imbalanced, which makes the performance of the models not very well. Studies on further optimizing the models to improve diagnostic performance to bridge the gap with PET/CT are worth looking forward to.

**Table 5:** Related studies of ML models based on different imaging features in distinguishing benign and malignant pulmonary nodules.

Studies	Sample size	Feature quantity	Feature type	Best model	AUC	Accuracy	Sensitivity	Specificity
Ma et al.^18^	1199	7	CT imaging features	Decision tree	0.746	–	0.762	0.799
Uthoff et al.^19^	327	18	CT imaging features, clinical features	Ensemble of neural networks	0.790	–	–	–
Beig et al.^20^	290	12	CT radiomic features	SVM	0.800	0.710	0.740	0.680
Zhang et al.^21^	82	4	PET/CT texture features	SVM	0.854	0.850	0.889	0.818
Chen et al.^22^	85	5	PET/CT texture features	SVM	0.910	0.860	0.840	0.910
Our proposed method	250	10	DLCT parameters, clinical features	LR	0.812	0.813	0.750	0.791

The performance of the models is inseparable from the extracted and filtered features. Among the 10 features selected through LASSO in this study, in addition to the clinical parameters of patients and the diameter of lesions, Δ_70 keV_, CT_SPN_70 keV_, CT_SPN_40 keV_, CER_40 keV_ and Δ_SA_70 keV_ are the parameters related to CT values, while CaS_SPN_ is the parameter related to energy spectrum values. Among them, Δ_70 keV_ is a parameter derived from CT_SPN_70 keV_. Previous studies have reported that the reconstruction of 70 keV monoenergetic images is roughly equivalent to standard dual-energy CT acquisitions performed at 120 kVp^23,24^. Therefore, CT_SPN_70 keV_ corresponds to the standard routine CT values, and Δ_70 keV_ corresponds to the standard enhancement levels. In the results of this study, CT_SPN_70 keV_ and Δ_70 keV_ in the SPN malignant group were significantly higher than those in the benign group, which was consistent with previous studies of conventional CT and energy spectrum CT in the differential diagnosis of benign and malignant pulmonary nodules^17,25,26^. The monoenergetic images with low keV can improve the contrast between iodine-containing pulmonary nodules and surrounding tissues, while CT_SPN_40 keV_, as the CT values measured by SPN on 40 keV monoenergetic images, can reflect the degree of enhancement of pulmonary nodules under 40 keV monoenergetic images, so it can indirectly reflect the blood supply in the lesions. Many previous studies have shown that the CT values of lesions on 40 keV monoenergetic images can distinguish benign and malignant lesions^16,27,28^. In a study by Zhang et al. to differentiate between solitary lung adenocarcinoma and pulmonary tuberculosis, solitary lung adenocarcinoma had a higher CT_40 keV_ value in the arterial phase^29^. In our study, higher levels of CT_40 keV_ values also predicted increased malignancy of SPN. The reasons may be that compared with benign SPNs, malignant SPNs have increased release of angiogenic factors, causing an increase in microvessel density range, which in turn increases capillary perfusion and permeability, leading to malignant nodules taking up more contrast media^25^. CER_40 keV_ and Δ_SA_70 keV_ are parameters derived from CT_SPN_40 keV_ and CT_SPN_70 keV_ respectively, and CaS_SPN_ is a DLCT quantitative parameter extracted from SPN. These three parameters were not statistically significant in this study, but they played an indispensable role in the ML models, which needs to be further discussed in larger research samples.

However, this study has some limitations. First, our data were retrospective, all data came from one center and lacked external verification, which limits the promotion of our models, and the results need to be verified in multicenter and external cohorts. In addition, there were few benign SPN data in this study, and there was a problem of data imbalance between the benign and malignant SPN groups. Although the synthetic minority oversampling technique was used to make the number of benign and malignant samples consistent, it may still affect the prediction performance of the models.

## Methods

### Patients

SPN patients were collected retrospectively at the Department of Thoracic Surgery of Jiangsu Cancer Hospital from September 2021 to March 2023. The inclusion criteria of this study subjects were as follows: ① benign and malignant SPNs confirmed by histopathology after surgery or biopsy and ② preoperative DLCT chest enhancement examination. The exclusion criteria were as follows: ① pure ground glass or subsolid nodules (containing ground glass components) (n = 11); ② a history of malignant tumors (n = 5); ③ poor CT image quality due to the lack of continuous thin-layer images or artifacts (n = 7); ④ the number of pulmonary nodules is more than one or primary nodules with several scattered lesions (n = 13); ⑤ a history of clinical antitumor treatment (n = 16); ⑥ lesions smaller than 10 mm (n = 8) and ⑦ nodules with poorly defined boundaries leading to poor segmentation (n = 4). After screening patients according to the above criteria, 250 patients with SPN were eventually enrolled in this study for follow-up analysis.

This retrospective study was approved by the Ethics Committee of Jiangsu Cancer Hospital (ethics number: 2023-048), and the experiment was conducted in strict accordance with the ethical standards set out in the 1964 Declaration of Helsinki and its subsequent amendments. The Ethics Committee of Jiangsu Cancer Hospital waived the written informed consent of the patients.

### DLCT image acquisition

All patients with SPN were examined by DLCT (IQon, Philips Healthcare, Best, The Netherlands), and breathing training was performed on each patient before scanning. With the patients lying supine on the scanning table, scan from the thoracic inlet to the bottom of the chest to cover all lung tissue. The contrast agent (ioversol, iodine 350 mg/mL, Hengrui Medicine, Lianyungang, China) was injected into the right elbow vein, and then the tube was flushed with 20 ml of normal saline. The injection rate was in the range of 2.5–3.0 ml/s, and the image acquisition during the enhancement period was delayed by 50 s after the injection was completed. The scanning slice thickness of all images was 5 mm and the reconstructed slice thickness was 1 mm. Other parameters were as follows: matrix, 512 × 512; collimator width, 64 × 0.625 mm; tube current automatic modulation; rotation time, 0.50 s; tube voltage, 120 kVp; scanning field of view, 372 mm; pitch, 0.900.

### DLCT image quantitative features

**Figure 3:**
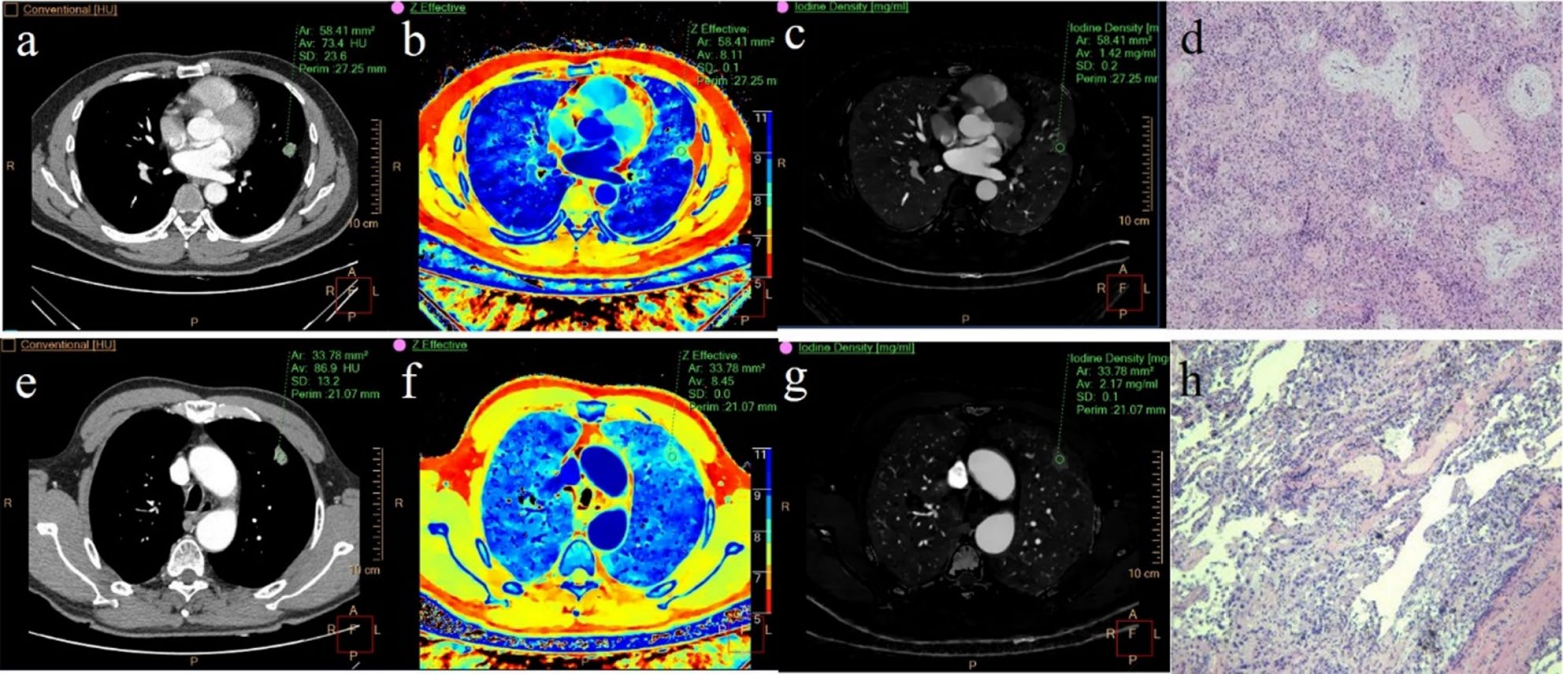
A 41-year-old male patient with inflammatory pseudotumor in the upper lobe of the left lung (**a**–**d**) and a 64-year-old male patient with lung adenocarcinoma (**e**–**h**), both pathologically confirmed. **a** and **e** are enhanced phase CT images, and the CT values of the lesions are 73.4 HU and 86.9 HU respectively; (**b**, **f**) are Zeff images with Zeff values of 8.11 and 8.45 respectively; (**c**, **g**) are iodine images, and the IC values are 1.42 mg/ml and 2.17 mg/ml respectively; (**d**, **h**) are postoperative pathological pictures.

All images were processed and analyzed on the Philips workstation (IntelliSpace Portal, Philips Healthcare). A radiologist used the workstation's built-in software (Spectral CT Viewer, Philips Healthcare) to delineate the circular regions of interest (ROIs) in the mediastinal window images and perform quantitative analysis, while another senior radiologist supervised from the sidelines. Before the analysis, neither doctor was informed of the clinical data and the pathological diagnosis results of benign and malignant SPN. Two examples (one SPN benign and the other SPN malignant) are shown in Fig. 3. ROIs should cover the areas of lesions with uniform density on the enhanced images to the greatest possible extent, avoiding calcification, blood vessels and necrotic areas. To ensure the stability of the results, the ROIs were drawn on the largest layer of the lesion cross-section and the layers above and below it, and the average of the three measurements was taken as the final analysis data. At the same time, a circular ROI was placed on the aorta with the largest cross-section of the lesion for the standardization of quantitative parameters. Then, other quantitative parameters of the same ROI were obtained on the VNC images, Zeff images, IC images, ED images, CaS images, and 40 keV and 70 keV monoenergetic images.

The quantitative parameters obtained from the ROIs delineated on the lesions and aorta were as follows: CT_SPN_VNC_, CT_SPN_40 keV_, CT_SPN_70 keV_, CT values of aorta on VNC images (CT_aorta_VNC_) and CT values of 70 keV after aortic enhancement (CT_aorta_). Derived parameters were calculated based on the above values, including SAR_VNC_, SAR_40 keV_, SAR_70 keV_, Δ_40 keV_, Δ_70 keV_, Δ_SA_40 keV_, Δ_SA_70 keV_, CER_40 keV_, CER_70 keV_, NEF_40 keV_, NEF_70 keV_ and λ_HU_. The calculation formulas of the above derived parameters were as follows^30–34^:

$${\text{SAR}}_{{{\text{VNC}}}} = {\text{ CT}}_{{{\text{SPN}}\_{\text{VNC}}}} /{\text{CT}}_{{{\text{aorta}}\_{\text{VNC}}}}$$

$${\text{SAR}}_{{{4}0\;{\text{keV}}}} = {\text{ CT}}_{{{\text{SPN}}\_{4}0\;{\text{keV}}}} /{\text{CT}}_{{{\text{aorta}}}}$$

$${\text{SAR}}_{{{7}0\;{\text{keV}}}} = {\text{CT}}_{{{\text{SPN}}\_{7}0\;{\text{keV}}}} /{\text{CT}}_{{{\text{aorta}}}}$$

$$\Delta_{{{4}0\;{\text{keV}}}} = {\text{ CT}}_{{{\text{SPN}}\_{4}0\;{\text{keV}}}} {-}{\text{CT}}_{{{\text{SPN}}\_{\text{VNC}}}}$$

$$\Delta_{{{7}0\;{\text{keV}}}} = {\text{CT}}_{{{\text{SPN}}\_{7}0\;{\text{keV}}}} {-}{\text{CT}}_{{{\text{SPN}}\_{\text{VNC}}}}$$

$$\Delta_{{{\text{SA}}\_{4}0\;{\text{keV}}}} = {\text{CT}}_{{{\text{SPN}}\_{4}0\;{\text{keV}}}} {-}{\text{CT}}_{{{\text{aorta}}}}$$

$$\Delta_{{{\text{SA}}\_{7}0\;{\text{keV}}}} = {\text{CT}}_{{{\text{SPN}}\_{7}0\;{\text{keV}}}} {-}{\text{CT}}_{{{\text{aorta}}}}$$

$${\text{CER}}_{{{4}0\;{\text{keV}}}} = \Delta_{{{4}0\;{\text{keV}}}} /{\text{CT}}_{{{\text{SPN}}\_{\text{VNC}}}}$$

$${\text{CER}}_{{{7}0\;{\text{keV}}}} = \Delta_{{{7}0\;{\text{keV}}}} /{\text{CT}}_{{{\text{SPN}}\_{\text{VNC}}}}$$

$${\text{NEF}}_{{{4}0\;{\text{keV}}}} = \Delta_{{{4}0\;{\text{keV}}}} /\left( {{\text{CT}}_{{{\text{aorta}}}} {-}{\text{CT}}_{{{\text{aorta}}\_{\text{VNC}}}} } \right)$$

$${\text{NEF}}_{{{7}0\;{\text{keV}}}} = \Delta_{{{7}0\;{\text{keV}}}} /\left( {{\text{CT}}_{{{\text{aorta}}}} {-}{\text{CT}}_{{{\text{aorta}}\_{\text{VNC}}}} } \right)$$

$$\uplambda _{{{\text{HU}}}} = \left( {{\text{CT}}_{{{\text{SPN}}\_{4}0\;{\text{keV}}}} {-}{\text{CT}}_{{{\text{SPN}}\_{7}0\;{\text{keV}}}} } \right)/\left( {{7}0{-}{4}0} \right)$$

Considering the differences in cardiac function and hemodynamics between patients, the IC automatically measured on the iodine image was normalized to the aorta, the NIC was calculated, and the NCaS, NED and NZeff were calculated in the same way. The calculation formulas were as follows:

$${\text{NIC}} = {\text{IC}}_{{{\text{SPN}}}} /{\text{IC}}_{{{\text{aorta}}}}$$

$${\text{NCaS}} = {\text{CaS}}_{{{\text{SPN}}}} /{\text{CaS}}_{{{\text{aorta}}}}$$

$${\text{NED}} = {\text{ED}}_{{{\text{SPN}}}} /{\text{ED}}_{{{\text{aorta}}}}$$

$${\text{NZeff}} = {\text{Zeff}}_{{{\text{SPN}}}} /{\text{Zeff}}_{{{\text{aorta}}}}$$

### Machine learning model

All SPN patients were randomly divided into the training and test sets by 7꞉3 and the patients in the training set were balanced at a ratio of 1:1 by using the synthetic minority oversampling technique, so that the number of benign SPN patients and malignant SPN patients was consistent^35,36^. The LASSO algorithm was used to screen features from patients’ clinical data and DLCT parameters, and 6 classical ML models were constructed in the training set based on the selected features, namely AdaBoost, GNB, LR, RF, SVM and XGBoost^37^. The ROC curves of the 6 models were plotted to obtain the AUC of the respective models, and the accuracy, sensitivity and specificity were calculated simultaneously to serve as the evaluation indicators for the 6 models. The models were evaluated by tenfold cross-validation, and the performance of the models was further validated using test set data. The above modeling process was based on R software (version 4.2.3) and Python programming language (version 3.11.4).

### Statistical analysis

The Shapiro–Wilk test was employed to assess the normal distribution of the data. The independent sample *t*-test was utilized for comparing the features of continuous data with a normal distribution, while the Mann–Whitney *U* test was used for comparing the features of continuous data with a non-normal distribution. The chi-square test or *Fisher’s* exact test was applied to compare the characteristics of count data. The statistical analysis of patients' clinical data and DLCT parameters was conducted using SPSS Statistics 26.0 (IBM Corp., Chicago, Illinois, United States of America) software, with statistical significance indicated by *P* < 0.05.

## Conclusion

When the DLCT quantitative parameters and derived parameters are combined with the ML models, the performance may be further improved in diagnosing benign and malignant SPNs, especially the LR model. By combining valuable DLCT parameters, the LR model can provide more diagnostic value for preoperative identification of benign and malignant SPNs, thereby helping clinicians make more reliable clinical decisions for the treatment of SPN patients. However, multicenter data are still needed for further confirmation. Furthermore, the biological mechanism underlying the relationship between DLCT parameters and benign and malignant SPNs requires further study.

## Data Availability

The datasets generated during and/or analyzed during the current study are available from the corresponding author on reasonable request.
